# Increasing participation of people with thought disorder in clinical research

**DOI:** 10.1192/j.eurpsy.2026.12211

**Published:** 2026-04-27

**Authors:** Lena Palaniyappan, Sylvain Baillet, Valentina Bambini, Etienne Barou-Laforie, Marta Bosia, Oli Delgaram-Nejad, Hashwin Ganesh, Ranjini Garani, Neil Harrison, Vegas Hodgins, Ridha Joober, Tilo Kircher, Gina Kuperberg, Chantal Murthy, Susan Rossell, Krish D. Singh, Iris E. Sommer, Sunny X. Tang, Debra Titone, Alban Voppel, Tosh Watson, Irnes Zeljkovic

**Affiliations:** 1 Department of Psychiatry, Douglas Mental Health University Institute, McGill University, Canada; 2 Centre de Recherche du Centre Hospitalier de l’Université de Montréal (CRCHUM) Département of Neurosciences, University of Montreal, Canada; 3 Neurolinguistics and Experimental Pragmatics Laboratory (NEPLab), IUSS: Scuola Universitaria Superiore Pavia, Italy; 4 Centre of Excellence in Youth Mental Health, Douglas Mental Health University Institute, Canada; 5 Vita-Salute San Raffaele University: Universita Vita Salute San Raffaele, Italy; 6 Machester Metropoliton University, UK; 7 School of Medicine, Cardiff University, UK; 8 Department of Psychology, McGill University, Canada; 9 Department of Psychiatry, Marburg University, Germany; 10 Department of Psychology, Tufts University and Massachusetts General Hospital, USA; 11 Department of Psychiatry, University of Washington, USA; 12 Centre for Mental Health, Swinburne University of Technology, Melbourne and InsideOut Institute, University of Sydney and Sydney Local Health District, Australia; 13 Cardiff University Brain Research Imaging Centre (CUBRIC), School of Psychology, Cardiff University, United Kingdom; 14 Department of Neuroscience, University Medical Center Groningen, The Netherlands; 15 Feinstein Institutes for Medical Research and Zucker School of Medicine, Northwell Health, USA

**Keywords:** clinical research inclusion, lived experience, severe mental illness, thought disorder, psychosis

## Abstract

**Background:**

Thought disorder (TD) is a core feature of severe mental illnesses such as schizophrenia, characterized by disruptions in speech, language, and communication. People with TD face unique barriers that hinder their involvement in research, both as participants and as partners. Their systematic underrepresentation in psychiatric research is driven by pervasive assumptions about their decisional capacity, willingness to participate, and ability to engage in research. This perpetuates a biased evidence base, likely hindering the therapeutic progress toward addressing this core problem.

**Methods:**

This review, informed by professional (clinical and research) and lived (bottom-up and phenomenological) experience of TD, examines how flawed assumptions regarding capacity, engagement, and participatory abilities serve as active barriers to inclusion.

**Results:**

We argue for a shift toward supported inclusion through tailored capacity assessments, enhanced informed consent procedures, targeted training of research personnel, and systemic institutional practices. Incorporating lived experiences of those with TD as research partners is integral to this approach, fostering co-production of research that is more valid, inclusive, and applicable.

**Conclusions:**

Without these inclusion-focused changes, the development of treatments for TD is likely to have very slow progress and a critical segment of the severely unwell population will continue to be underrepresented from the scientific process, undermining both the utility and generalizability of psychiatric research.

## Introduction

Thought disorder (TD, also called Formal Thought Disorder or FTD) is a clinically significant symptom dimension of many severe mental illnesses (SMI) [1, 2]. It presents in two broad forms: disorganization (positive TD), characterized by incoherent, illogical, or tangential speech with difficulty maintaining goal-directed discourse; and impoverishment (negative TD), characterized by reduced speech quantity, poverty of content, and slowed verbal output. TD affects both verbal and nonverbal communicative behavior, and is particularly common in people with psychotic disorders (50–70% in some cohorts [3, 4]) and mood disorders [2, 5, 6]. TD often precedes and predicts episodic psychotic symptoms (e.g., hearing voices, paranoia) [7, 8], yet can persist despite treatment and significantly increase the risk of relapse [9, 10]. The presence of TD typically indicates higher illness severity [11, 12].

TD is a neglected therapeutic target, with no treatment that specifically addresses TD once it is diagnosed. While TD symptoms (especially disorganization) in the early phases of illness may improve with targeted multimodal early intervention [13], over the longer term, both negative and positive TD often persists despite the remission of other psychotic symptoms, contributing to long-term functional impairment and poorer clinical outcomes [14–17]. Evidence suggests that decline in cognitive, vocational, and functional outcomes [18–21] as well as the disrupted sense of self [22] that characterizes illnesses such as schizophrenia are predominantly influenced by persistent TD. Reducing the burden of TD could potentially mitigate social exclusion [23] and improve quality of life [20].

Several promising preliminary findings targeting communication impairments suggest the potential for targeted intervention [24–27]. However, large-scale trials to understand therapeutic targets (i.e., the cognitive and mechanistic processes behind TD) and test potential treatments are lacking. The measurement of TD per se varies considerably in current practice [28, 29], with the resulting operational heterogeneity itself being a barrier to cumulative knowledge about the participation of people with TD. More importantly, lived experience perspectives remain largely absent in TD research; it is this aspect that we focus on in the current work. We argue that TD research is paradoxically hindered by the systematic, often unexamined noninclusion of those most affected by it, resulting in a biased evidence base that perpetuates therapeutic nihilism. Given the prevalence and pervasiveness of TD in SMI, closing this inclusivity gap is key both to understanding its pathophysiology and to improving overall recovery rates in SMI.

## Methodology

We employed a qualitative, experienced-informed methodological approach to examine how current research practices shape the inclusion of individuals with SMI, with a particular focus on those experiencing TD. This approach, grounded in collaborative expertise, integrated two distinct knowledge sources: combined professional research experience (from coauthors with backgrounds in psycholinguistics, psychiatry, neuroimaging, cognitive neuroscience, and clinical trials) and lived experience (from coauthors and network members with personal experience of TD and its impact on social and research participation). This enabled a combined epistemic perspective on how assumptions about capacity, willingness, and communication influence recruitment, consent, and study design processes.

We collected evidence through three methods: (1) Reviewing published literature on eligibility, recruitment, and consent practices in SMI research. Searches were conducted in PubMed by combining variations of the terms (“thought disorder” OR “formal thought disorder” OR “disorganisation” OR “impoverished speech”) with (“clinical trial” OR “research participation” OR “informed consent” OR “decisional capacity” OR “recruitment” OR “exclusion criteria”). Reference lists of included papers were hand searched. No date restriction was applied; publications up to December 2025 were considered. (2) Assessing the alignment of identified practices with empirical evidence on decisional capacity, motivation, and communicative variability in TD, and (3) identifying areas for strengthening current practices and formulating recommendations to support inclusion. These recommendations were iteratively refined through structured discussion with individuals with lived experience of TD, within the DISCOURSE in Psychosis Network, and validated against existing consent and capacity enhancement literature. The resulting recommendations reflect this integrated epistemic process rather than a formal systematic review; we use the term “experienced-informed review” to distinguish this approach. We additionally identified areas in which current practices could be strengthened and formulated corresponding recommendations to enhance supported participation.

## Results

Exclusion of people with TD from clinical research operates through at least three interlocking mechanisms. First, ethical and regulatory barriers arise from concerns (often assumed rather than assessed) about decisional capacity and vulnerability, and lead to explicit protocol-level exclusion criteria. Second, methodological barriers emerge from design choices that inadvertently disadvantage people with TD, such as requirements for sustained digital engagement or complex language comprehension. Third, logistical and pragmatic barriers reflect clinician workload, unfamiliarity with supported consent procedures, and implicit gatekeeping (constituting what we term assumptive exclusion below). All three drivers interact and frequently reinforce one another.

Individuals with SMI are generally willing to participate in research, often for altruistic reasons [30, 31]. Nevertheless, many patients likely including those with notable TD – are explicitly excluded from clinical trials [32] and many more are never approached. Only one in five “real-world” patients with schizophrenia appear eligible based on explicit exclusion criteria for randomized efficacy [33] and effectiveness [34] trials. This is worsened by symptom-based exclusion criteria for emerging interventions recruiting community-living patients (e.g., more than minimal disorganization for digital therapeutics [35, 36]) that disproportionately affect people with TD. More damagingly, most eligible patients never get invited to participate [37]. For example, although 20% of people with psychosis had ever been approached to consent to contact by clinicians [38], 65% of those approached agreed to enter a research register; those left out are likely to have a higher burden of TD.

Multiple implicit factors based on subjective clinical impressions shape who gets invited into clinical research. The resulting unacknowledged exclusions constitute “disappearing participants” (e.g., people with cognitive impairment in geriatric research) [39]. Compared with the rest of the broader clinical population with psychosis, excluded patients show poorer therapeutic engagement, impaired cognition [40, 41], and lower psychosocial functioning [40, 42, 43]. All of these factors – poor psychosocial functioning [20, 41–44], impaired cognition [45–49], and poor engagement [50] – are more strongly influenced by TD than by other symptoms of psychosis. The most direct evidence for TD-specific gatekeeping comes from Riedel and colleagues who noted that people with TD were seven times more likely to be excluded from trials testing antipsychotic efficacy [42] – a ratio not explained by overall illness severity. Lally and colleagues identified “cognitive problems” including incoherent speech were more among people not invited to a lifestyle intervention trial [40].

Beyond interventional trials, the consequences of selective exclusion extend beyond representativeness to compromise both external validity and statistical power. Not approaching people with TD has substantial implications for studies that aim to understand neurobiological mechanisms per se. For example, in the largest and most well-powered study of the neuroanatomy of TD (*n* = 2008), the median score on the primary TD measure (PANSS P2; scale range 1–7) was 2 (interquartile range: 1–3; patients *n* = 752), indicating that most patients had no or clinically insignificant TD [51]. This low TD score is in keeping with the overall low symptom burden, but highlights the lack of representativeness of people with notable TD even in TD-specific mechanistic studies. The resulting estimates of neural correlates, derived from a range-restricted sample, may not capture the pathophysiological signal most relevant to TD. This attenuates the true effect, inflates required sample sizes to the detriment of further research efforts, and limits generalizability to the population for whom therapeutic targets are most urgently needed.

**Figure 1.**
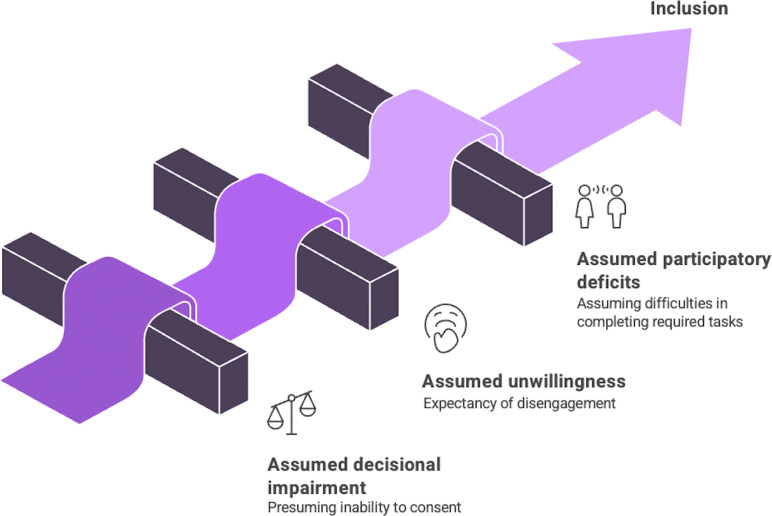
Assumptive exclusion affecting the enrolment of people with thought disorder in clinical research arises from flawed assumptions about capacity, willingness, and abilities. Figure made using napkin.ai.

To make clinical research in SMI truly generalizable, we should address the biased practices that lead to the systematic exclusion of people with TD. Based on our professional research experience and lived experience, we find the key barriers are not evidence based but stem from flawed assumptions about capacity, willingness, and communication. We call this phenomenon *assumptive exclusion*, which creates several major, and often intersecting, barriers to participation that we describe next (Figure 1).

Box 1: A supported inclusion package recommended as a basic minimum for studies recruiting people with SMI and thought disorder (TD)

**Tier 1 (Essential: applicable to all studies):**

- Replace diagnosis-based exclusion with function-based assessment.
- Use simplified consent materials (e.g., for English, Flesch–Kincaid Grade ≤7 and equivalent readability for other languages; short sentences; no metaphorical/abstract language in information sheets; visual/multimedia aids).
- Administer a brief standardized capacity screen (e.g., UBACC) for participants meeting the trigger criteria outlined in Section “Incorporate capacity assessment”.
- Train all research personnel in TD-aware communication (minimum: 2-hour session covering assessment and accommodation strategies).
- Always include a lived experience consultant in study design and to review materials.
- Designate a single study point of contact throughout the study period.

**Tier 2 (Enhanced: for studies involving complex/high-risk procedures):**

- Administer full capacity assessment if capacity is uncertain after Tier 1 screen.
- Offer alternative consent formats: pre-recorded video summary; FAQ prompt sheet; advanced consent window.
- Document and report accommodations used (to improve the evidence base for supported inclusion).
- Allow multiple consent sessions and permit a support person.

## Assumptive exclusion

### Assumed lack of decision-making capacity

People with SMI, including those with TD, are often excluded from research on the basis of assumed impairments in decision-making capacity (DMC) for research. Patients are frequently perceived to lack capacity, even when they are capable [52]. This perceived actual capacity gap has resulted in unjustified exclusion of people with SMI across various trials [32, 53, 54]. Disorganization (positive TD) is most likely to impede comprehension of consent materials and underpin perceptions of poor communicative competence. Several studies have established that diagnostic labels such as disorganized schizophrenia should not be automatically linked to decisional impairment for research [55–60]. Although symptoms such as TD can influence DMC [56, 61–63], most studies in a systematic review of association between symptoms and research-related decisional impairment reported no or only weak correlations [55]. Thus, as with diagnostic labels, no single symptom, including TD, should be taken to indicate impaired research decisional capacity (see also Appelbaum [64] and Dunn [65]). In contrast, cognitive impairment, particularly short-term memory deficits [66–68], is a stronger predictor of decisional impairment than TD [55, 64, 68–72]. Importantly, these memory difficulties are remediable [68, 69], and once addressed, capacity in patients with schizophrenia can be retained over time [69, 73]. Decisional impairment for research is best considered “temporal, identifiable, and responsive to interventions” [74].

People with TD are likely to be subjected to compulsory treatments [75] on the basis of poor disease insight [76–78], higher risk of treatment nonadherence [79], and reduced DMC for treatment [80]. Clinicians and researchers are often unwilling to approach the compulsorily treated people for research studies on the assumption of absent research DMC [81, 82]. Such heuristics are problematic as the cognitive and functional demands of consenting to research may differ from those required for treatment decisions. In particular, studies have shown that a greater proportion of acute and severely unwell patients with schizophrenia retain research DMC, even when they lack treatment DMC (51% vs. 31% in a head-to-head comparison [56]). A critical difference between the two concepts is the role of disease insight. Unlike the decision to accept a proposed treatment, the decision to take part in a research study need not require one’s awareness, acceptance, and in many cases, the attribution of symptoms to an illness label. In empirical studies, insight has emerged as the key determinant of treatment decisions [55, 74, 83, 84], but does not play a prominent role in research decisions [55, 56]. In contrast, the ability to retain novel study-related information over a period of time is more critical for research-related decisional impairment [56]. Thus, blanket presumption of impaired research capacity due to involuntary treatment status is not supported by extant literature.

### Assumed unwillingness to participate

Clinicians often perceive people with TD to have poor therapeutic engagement [85, 86]; people with TD are often excluded from therapies where such engagement is a key ingredient (e.g., cognitive behavioral therapy [87], group metacognitive training [88]) even if these are likely to benefit TD [89, 90]. People with impoverished speech (negative TD) are more likely to limit expressive participation, creating the misleading impression of unwillingness. These assumptions, part of “first impressions” [91], may deter clinicians from recruitment efforts. When approached, people with SMI agree to take part in research studies in comparable numbers to other patient groups [38, 92–95], due to altruism and an expectancy of psychosocial benefits [30, 31, 96, 97]. Illness severity shows no associations with perceived benefits [98] and only modest associations with perceived harms [97]. Candillis and colleagues noted that people with schizophrenia unwilling to participate in a hypothetical drug trial had comparable severity of TD to a group that was willing to take part [99], refuting the assumption that TD signals unwillingness. Wu and colleagues observed more TD burden among patients with a higher willingness to participate in a hypothetical treatment study, likely due to the higher relevance of the benefits of the proposed treatment (a hypothetical agent for memory enhancement) [100]. Unlike research decisional impairment, willingness to participate does not vary with cognitive functioning [100, 101]; instead, it depends on the patient’s perceived risks of participation [30, 101–103] and the presence of research safeguards [104–106]. The assumption that people with TD may be unwilling for research has no empirical support.

### Assumed lack of participatory ability

People with TD, owing to their speech and language disturbances (both positive and negative TD), may have low participation in life situations in which knowledge, ideas, or feelings are exchanged [107, 108]. Because participation is central to research endeavors, recruiters may implicitly exclude such individuals. However, the relationship between TD and communicative participation is not fixed. Patients with TD who have participated in linguistic research describe the experience as validating and meaningful, irrespective of TD severity [109]. Many aspects of language comprehension, alignment, and communicative functioning are relatively preserved in TD (109–113) [110–114]. While complex language (e.g., figurative language [115] and grammatically complex sentences [116]) poses difficulties for some patients, core comprehension remains intact for many. Furthermore, impaired syntactic comprehension shows high variability, with many people demonstrating strong receptive language skills despite disorganized or reduced speech. TD can also fluctuate; communicative impairments vary over time, influenced by emotional states [117], and by relapsing/remitting phases of illness [10, 118, 119]. This episodic course distinguishes TD from major neurocognitive disorders or aphasia and creates opportunities to mitigate communication challenges, as we discuss later.

In summary, the practice of assumptive exclusion explains the substantially reduced inclusivity of people with TD in clinical research. These assumptions, combined with workloads [120] and resource constraints [121], result in substantial discretion in patient selection, turning recruitment of people with TD into a street-level bureaucracy [122]. To address this, we propose moving from a deficit-based model that excludes, to a support-oriented framework that adapts, includes, and finally represents this critical population. The accommodations described next are designed to address barriers arising from either dimension of TD, recognizing that they often coexist in the same individual and that the relative burden may fluctuate over time. Where these dimensions differ in their implications for specific recommendations, this is noted.

## Discussion

Our framework to improve the inclusion of people with TD as participants in research studies on SMI is centered on explicit capacity assessment (addressing 4.1), enhanced consenting and information exchange (addressing 4.2), targeted training/education (addressing 4.3), and reflexive institutional practices (a systemic solution).

### Incorporate capacity assessment

A number of empirical studies have explored the means by which decisional capacity for research can be assessed [123, 124] and enhanced [64, 65, 70, 125, 126] for people with SMI who are likely to have TD. To avoid the considerable variations in inferring decisional impairment based on professional intuition [127–129], a brief, study-specific assessment should be incorporated in the informed consent process [130] (for a detailed review of various instruments, see [124]). One notable resource is the UCSD Brief Assessment of Capacity to Consent (UBACC [131]), a brief (5–10 min), well-validated tool that covers both understanding and appreciation aspects of decision making [132]). Importantly, assessors (e.g., research assistants) must be trained in administering the brief tools [133]. To reduce assessment burden, the short research capacity assessment could be reserved for situations where decisional impairment for research is suspected rather than presuming incapacity based on diagnosis.

To operationalize “suspected decisional impairment for research” without reproducing subjectivity, we propose one of the following conditions to be met in order to trigger a study-specific capacity assessment: (1) the participant is currently subject to involuntary psychiatric treatment; (2) the participant is unable to comprehend some of the instructions in the most recent clinical encounter; or (3) the participant has been assessed to have decisional impairment for an unrelated task (e.g., financial decisions). Importantly, none of these triggers constitutes grounds for automatic exclusion; they are grounds for structured assessment. Routinely practicing this for all studies involving people with TD could explicitly remind clinicians of the sliding scale notion of capacity [134], that is, decisional capacity is not a fixed trait but varies with the nature and complexity of the proposed procedures.

### Enhanced consenting and information exchange

The “understanding” component of research decisional capacity is the most affected element in TD (especially in positive TD), and lack of retention of information is seen as the key cognitive problem behind this failure [56]. The use of enhancements, that is, aids to simplify (e.g., multimedia cues [135], improving comprehension [136]) and reinforce the key procedural information (e.g., via repetition [72, 137], iterative feedback [138]) has been shown to be of benefit (also see [65, 139, 140] for a review). Shorter and more readable consent forms with simple illustrations that are easy to read, providing information using short and grammatically simple sentences, avoiding ambiguities and metaphorical expressions, and enabling the use of participants’ dominant language [141] are essential general principles to overcome TD-related syntactic and pragmatic comprehension issues (see Supplementary Appendix 1 for an example). Reading ease of most consent forms appears to be poor [142]; intentional “easing” of the text materials used in clinical research (e.g., using participant feedback, lived experience experts’ input [143], and employing readability indices [144]) is required to achieve this goal. Options such as advanced consent when TD burden is low, consent to contact at the time of program entry, or proxy consenting [145] (including the use of subject advocates [146]) when TD is high may be an option for certain studies, though this may be feasible only for studies that rely on routinely collected information, rather than those involving active data-giving exercises.

An extensive review of various interventions to enhance research decisional capacity concluded that the key ingredient is the allocation of more one-to-one interaction time [125]. In particular, for patients with TD and impoverished speech (reduced verbal output, often monosyllabic and slow responses that are not elaborate and spontaneous), conversational convergence can be achieved if there is sufficient time for an interaction (see Hodgins and colleagues for further elaboration [147]). Providing a set of questions that are expected in a specific context (i.e., prompts or cues) is a technique used as part of social skills training in the presence of TD [148–151]; this approach of “frequently asked questions” can be used for consenting procedures to increase engagement (Supplementary Material S1). Such communication accommodations are important because informed consent is not a yes or no response; it is a process rather than a document (note similar considerations in aphasia [152, 153] and autism research). Providing pre-recorded information about key procedures in simple language that can be accessed at participants’ own pace (e.g., see Supplementary Material S2 – video link) also increases the contextual information that facilitates exchanges in the presence of impoverished speech [24]. Study teams should be able to offer more than one session for explaining research procedures, allow for accompanying persons if required, and be flexible to schedule them during periods when participants are less affected by TD (e.g., not immediately after hospitalization). These accommodations need to be clearly communicated to those who make the first contact for recruitment. A more detailed list of these approaches, adapted from the recommendations made by Working to increase Inclusivity in Research Ethics [154] can be found in Table 1.

**Table 1.** Recommendations for enhanced consenting in studies focused on TD

Recommendations	Rationale
Use short words and simple sentences that break down complex procedures in written materials, e.g., English – aiming for a Flesch–Kincaid Grade Level of ≤7, which corresponds to reading age of approximately 12–13 years.	Reduces cognitive load and aids retention, improving “understanding” aspect of decision making in the presence of TD.
Structure information in a logical, predictable order. Use clear headings like: “Who is doing this study?,” “What will I do?,” “What are the risks?”	Reduces confusion arising from referential impairments in TD.
Integrate visuals and icons. Use images to represent study steps, key concepts (e.g., a lock for confidentiality), and team members.	Supports comprehension through a nonlinguistic channel that may be less affected by TD. Provides a visual anchor for verbal information.
Avoid metaphors, idioms, and vague language. For example, instead of “we will let you know soon” use “we will call you in a week.”	Addresses TD-related impairment in the interpretation of nonliteral language.
Define necessary technical terms with simple examples. For example, “We will use an EEG. This is a cap with sensors that measures your brain’s activity.”	Makes abstract or unfamiliar concepts tangible and understandable, bridging the gap in jargon comprehension.
Allocate more one-to-one interaction time. Plan for multiple, shorter sessions to explain the study.	Facilitates participation in the presence of impoverished speech.
Flexibly schedule consenting sessions during periods of lower TD burden (e.g., avoid times of high stress or immediately after hospitalization).	Acknowledges the episodic nature of TD.
Provide information in advance and in multiple formats (e.g., pre-recorded video explaining the study, long and short formats).	Allows participants to review information at their own pace and through their preferred modality, aiding comprehension and retention.
Implement safeguards in advance and in multiple formats (e.g., no coercion, possibility to interrupt, data privacy).	Allows participants to engage on their own terms, pause or withdraw at any point, and ensures understanding while protecting privacy and autonomy.
Use conversational prompts and cues (e.g., a FAQ list of questions participants might want to ask). Use interactive questions to check understanding.	Supports the social-pragmatic aspect of communication. Provides a structure for the conversation, reducing the demand on expressive language.
Allow for a support person to be present during consent discussions.	Provides communicative support and helps to obtain clarifying information.
Implement a single point of contact throughout the study.	Builds trust and reduces the cognitive demand of having to re-explain one’s situation to new people.
Use study-specific consent checklists instead of rigid templates.	Facilitates incorporating the above recommendations.
Use nonverbal cues, simple nonmetaphoric gestures, tone of voice, and facial expressions, but do not overdo it.	Use more channels other than the verbal one.
Let the participant summarize information in his or her own words in smaller pieces.	To check whether there is mutual understanding.

### Targeted training

Investing in training research staff on capacity assessment, open engagement, and visibility in clinical spaces has significantly improved consent procedures and successfully recruited a conventionally excluded sample of continuously hospitalized patients with SMI for a genetic study [82]. In addition, to directly counter the assumptive exclusion, targeted sessions can be planned for both frontline clinicians and research teams throughout the study period. Clinicians often rely on subjective impressions of TD due to limited linguistic training and time constraints, leading to inconsistent assessments of communicative ability.

Integrating structured TD assessment tools and training, co-developed with lived experience experts, must move beyond deficit focus to practical, evidence-based skills to foster engagement in the presence of TD (see [50, 155] for example).

### Institutional accommodations

Institutional Review/Ethics Boards often mandate that patients with SMI be recruited for research through initial contact by their treating clinicians. This approach is common across jurisdictions, with physicians making the first approach at many institutions [31, 103, 156]. Some study designs rely on treating clinicians to assess eligibility [157] or to identify participants across the spectrum of TD [158, 159]. For such studies, collecting data as a by-product of routine healthcare visits, having a single point of contact throughout the study period, and sending text message reminders regarding research interviews may facilitate the participation of people with TD.

Relying on clinicians alone to identify participants with TD introduces two types of selection bias. First, TD is often an “invisible” problem as patients do not complain about being “thought disordered.” Subtle TD often goes undetected during brief clinical interactions, affecting studies that focus on people with TD identified via clinical referrals. On the other hand, as discussed earlier, assumptive exclusion precludes the more severely affected individuals from being referred. Reducing reliance on clinical referrals can potentially reduce selection bias [157, 160] that arises from the conflation of therapeutic engagement and factors related to therapeutic uncertainty [121] with research participation willingness [157] and ensure samples that are more representative of the full spectrum of TD. Institutional support for preconsent screening (see [161] for example) can remove this barrier, especially in the presence of electronic health record infrastructure and consent to contact procedures [38, 92]. Another approach is the use of “third-sector referrals,” for example, employment centers, community organizations, support groups, advocacy groups (e.g., see Bosco and colleagues [162]), and through past research participants (e.g., see Yoon and colleagues [158]).

Researchers anticipating ethics board concerns about including participants with TD should address the following explicitly in their protocols: (1) specify the capacity assessment instrument and triggers (as in Section “Incorporate capacity assessment”), demonstrating that inclusion is based on assessed rather than assumed capacity; (2) describe the consent accommodations planned (simplified materials, multiple sessions, supported consenting options) with reference to published evidence for their efficacy; (3) include a monitoring plan that is proportionate to the study risk and adapted for participants with TD (e.g., structured check-in calls using a single named contact rather than complex questionnaires); and (4) build the research governance team with lived experience advisors. At the institutional level, common barriers to implementing these approaches include variability in IRB/REC familiarity with supported consent procedures, limited staff time, and absence of reimbursement for additional consent sessions. We recommend that funders and institutions treat enhanced consent procedures as legitimate cost items in grant budgets, and that research ethics frameworks clarify that the use of supported consent is the preferred practice when recruiting people with SMI.

## The missing voices

An important lacuna in SMI research, in general, and TD research, in particular, is the missing voice of people with lived experience of TD in research studies. Despite the growing integration of lived experience expertise in mental health research elsewhere (e.g., see [163, 164]), most studies on TD do not involve people with lived experience of TD or loved ones/family members as research partners. Of 231 primary studies included in 7 systematic reviews focused on TD (1 on cognitive features [165], 2 on linguistic features [166], 2 on brain imaging [167, 168], and 2 on communication interventions [24, 27]), none reported active and explicit involvement of Lived Experience expertise in the design, implementation, and dissemination of research. Thus, at a time where coproduction of randomized trials is taking off at a rapid pace elsewhere [164, 169], and the concept of “nothing about us without us” [170] changing public perceptions about conditions such as autism [171], a seat at the tables where research is planned and produced remains aspirational for people with lived experience of TD (see Supplementary Material S3).

Coproducing SMI research with people having lived experience of TD requires infrastructure that fosters outreach beyond the conventional “clinic-based” models of psychiatric research [172]. This can lead to the selection of meaningful outcomes, especially for clinical trials, reduce assessment burden (e.g., using self-report instruments, recorded spontaneous narratives), enhance the consent process, reinforce relational power, and educate researchers and clinical teams about willingness to participate in research. The key ingredients for collaboration in this regard include the establishment of long-term local coproduction partnerships and platforms fostering experiential science. The experientially driven cowritten recommendations presented earlier emerged from one such collaboration fostered by the DISCOURSE in Psychosis network.

Meaningful partnership with people with lived experience of TD (PLE) should be embedded across the full research cycle, not treated as an after-thought or add-on. At the stage of priority setting, PLE can contribute to developing research questions that meaningfully address TD, ensuring that studies address burdens that matter to those most affected. During materials development, PLE input is directly linked to the consent tools described in this article: PLE can assess whether consent forms achieve the readability and clarity targets (Table 1) and codevelop the FAQ prompt sheets and video summaries described in Section “Enhanced consenting and information exchange”. At the recruitment stage, PLE can enhance outreach to ensure representative samples and engage with peer support groups or advocacy organizations – thus widening access beyond clinical referral pathways. In outcome selection, coproduction ensures that primary outcomes include measures of communicative participation and quality of life that are meaningful from a PLE perspective, and not only symptom assessments. Finally, at dissemination, PLE can help translate findings into accessible formats for patient communities, close the feedback loop, and advocate for the policy changes needed to sustain supported inclusion practices. These contributions require adequate resourcing: PLE should be remunerated fairly and supported with training and preparation time.

### Counterarguments

We acknowledge that there are contexts in which the non-inclusion of people with TD may be scientifically justified or ethically required. Where a trial outcome depends on a task that is incompatible with severe disorganization (e.g., paradigms requiring reliably organized verbal and nonverbal behavior), or where safety monitoring mandates real-time verbal communication that cannot be adequately supported in the presence of notable communicative impairment, exclusion may be appropriate. It will be appropriate for institutions and ethics committees to expect sound reasoning for this exclusion, and a consideration of alternatives that facilitate inclusion. The supported inclusion framework advocated here does not argue for inclusion *at any cost*; it argues that the current default of “exclusion without systematic assessment” should be reversed in favor of “inclusion *with appropriate* *support*,” and that the burden of justification should lie with exclusion, not inclusion.

## Conclusion

In conclusion, the systematic noninclusion of people with thought disorder from research is not a neutral oversight but an active barrier to scientific progress and therapeutic innovation. Not including their voices means missing out on unique experiential expertise and leads to less representative studies. To dismantle this barrier, we must move beyond flawed assumptions and replace assumptive exclusion with a framework of supported inclusion. By embracing enhanced consent procedures, reflexive institutional practices, and most critically, the leadership of those with lived experience, we can generate a more valid and equitable evidence base. But without the support of funders and institutions acknowledging the need for extra resources and flexibility, the proposed supported inclusion framework risks becoming another mandate that places moral responsibility on individual actors without dismantling the systemic disincentives that make assumptive exclusion the easier option. Ultimately, the path to understanding and effectively treating thought disorder is paved not by excluding those it affects, but by intentionally and respectfully including them.

## Supporting information

10.1192/j.eurpsy.2026.12211.sm001 Palaniyappan et al. supplementary material Palaniyappan et al. supplementary material

## Data Availability

No new data were generated or analyzed in this study. Data sharing is therefore not applicable to this article.

## Supplementary material

The supplementary material for this article can be found at http://doi.org/10.1192/j.eurpsy.2026.12211.

## References

[1] Palaniyappan L. Dissecting the neurobiology of linguistic disorganisation and impoverishment in schizophrenia. Semin Cell Dev Biol. 2022;129:47–60. 10.1016/j.semcdb.2021.08.015. 34507903

[2] Kircher T, Bröhl H, Meier F, Engelen J. Formal thought disorders: from phenomenology to neurobiology. Lancet Psychiatry. 2018;5:515–26. 10.1016/S2215-0366(18)30059-2. 29678679

[3] Breier A, Berg PH. The psychosis of schizophrenia: prevalence, response to atypical antipsychotics, and prediction of outcome. Biol Psychiatry. 1999;46:361–4. 10.1016/S0006-3223(99)00040-2. 10435201

[4] Cuesta MJ, Peralta V. Testing the hypothesis that formal thought disorders are severe mood disorders. Schizophr Bull. 2011;37:1136–46. 10.1093/schbul/sbr092. 21857008 PMC3196948

[5] Palaniyappan L, Wang Y. Disorganisation and depression: a re-examination of how we think and speak when depressed. Eur Arch Psychiatry Clin Neurosci. 2025; 10.1007/s00406-025-01994-1. 40172688

[6] Stein F, Lemmer G, Schmitt S, Brosch K, Meller T, Fischer E, et al. Factor analyses of multidimensional symptoms in a large group of patients with major depressive disorder, bipolar disorder, schizoaffective disorder and schizophrenia. Schizophr Res. 2020;218:38–47. 10.1016/j.schres.2020.03.011. 32192794

[7] Dominguez M-G, Saka MC, Lieb R, Wittchen H-U, van Os J. Early expression of negative/disorganized symptoms predicting psychotic experiences and subsequent clinical psychosis: a 10-year study. AJP. 2010;167:1075–82. 10.1176/appi.ajp.2010.09060883. 20634371

[8] Bearden CE, Wu KN, Caplan R, Cannon TD. Thought disorder and communication deviance as predictors of outcome in youth at clinical high risk for psychosis. J Am Acad Child Adolesc Psychiatry. 2011;50:669–80. 10.1016/j.jaac.2011.03.021. 21703494 PMC3124656

[9] Sandini C, Zöller D, Schneider M, Tarun A, Armando M, Nelson B, et al. Characterization and prediction of clinical pathways of vulnerability to psychosis through graph signal processing. elife. 2021;10:e59811. 10.7554/eLife.59811. 34569937 PMC8476129

[10] Häfner H, Maurer K, der Heiden W. ABC schizophrenia study: an overview of results since 1996. Soc Psychiatry Psychiatr Epidemiol. 2013;48:1021–31. 10.1007/s00127-013-0700-4. 23644725

[11] Oeztuerk OF, Pigoni A, Wenzel J, Haas SS, Popovic D, Ruef A, et al. The clinical relevance of formal thought disorder in the early stages of psychosis: results from the PRONIA study. Eur Arch Psychiatry Clin Neurosci. 2022;272:403–13. 10.1007/s00406-021-01327-y. 34535813 PMC8938366

[12] Roche E, Creed L, MacMahon D, Brennan D, Clarke M. The epidemiology and associated phenomenology of formal thought disorder: a systematic review. Schizophr Bull. 2015;41:951–62. 10.1093/schbul/sbu129. 25180313 PMC4466171

[13] Pelizza L, Leuci E, Maestri D, Quattrone E, Azzali S, Paulillo G, et al. Disorganization in first episode schizophrenia: treatment response and psychopathological findings from the 2-year follow-up of the “Parma early psychosis program. Journal of Psychiatric Research. 2021;141:293–300. 10.1016/j.jpsychires.2021.07.015. 34274840

[14] Brasso C, Bellino S, Bozzatello P, Del Favero E, Montemagni C, Rocca P. Inter-relationships among psychopathology, cognition, and real-life functioning in early and late phase schizophrenia: a network analysis approach. Schizophr Res. 2023;256:8–16. 10.1016/j.schres.2023.04.011. 37120939

[15] Comparelli A, Corigliano V, Forcina F, Bargagna P, Montalbani B, Falcone G, et al. The complex relationship among formal thought disorders, neurocognition, and functioning in nonacutely ill schizophrenia patients. J Nerv Ment Dis. 2020;208:48–55. 10.1097/NMD.0000000000001087. 31738225

[16] Roche E, Lyne J, O’Donoghue B, Segurado R, Behan C, Renwick L, et al. The prognostic value of formal thought disorder following first episode psychosis. Schizophr Res. 2016;178:29–34. 10.1016/j.schres.2016.09.017. 27639419

[17] Liddle PF. The Core deficit of classical schizophrenia: implications for predicting the functional outcome of psychotic illness and developing effective treatments. Can J Psychiatr. 2019;64:680–5. 10.1177/0706743719870515. PMC6783668 31434513

[18] Norman RMG, Malla AK, Cortese L, Cheng S, Diaz K, McIntosh E, et al. Symptoms and cognition as predictors of community functioning: a prospective analysis. AJP. 1999;156:400–5. 10.1176/ajp.156.3.400. 10080555

[19] Dickinson D, Bellack AS, Gold JM. Social/communication skills, cognition, and vocational functioning in schizophrenia. Schizophr Bull. 2007;33:1213–20. 10.1093/schbul/sbl067. 17164469 PMC2632341

[20] Rocca P, Galderisi S, Rossi A, Bertolino A, Rucci P, Gibertoni D, et al. Disorganization and real-world functioning in schizophrenia: results from the multicenter study of the Italian network for research on psychoses. Schizophr Res. 2018;201:105–12. 10.1016/j.schres.2018.06.003. 29898819

[21] Sigaudo M, Crivelli B, Castagna F, Giugiario M, Mingrone C, Montemagni C, et al. Quality of life in stable schizophrenia: the relative contributions of disorganization and cognitive dysfunction. Schizophr Res. 2014;153:196–203. 10.1016/j.schres.2014.01.013. 24485197

[22] Sass L, Parnas J. Thought disorder, subjectivity, and the self. Schizophr Bull. 2017;43:497–502. 10.1093/schbul/sbx032.

[23] de Sousa P, Sellwood W, Eldridge A, Bentall RP. The role of social isolation and social cognition in thought disorder. Psychiatry Res. 2018;269:56–63. 10.1016/j.psychres.2018.08.048. 30145302

[24] Jimeno N. Language and communication rehabilitation in patients with schizophrenia: a narrative review. Heliyon. 2024;10:e24897. 10.1016/j.heliyon.2024.e24897. 38312547 PMC10835363

[25] Bambini V, Agostoni G, Buonocore M, Tonini E, Bechi M, Ferri I, et al. It is time to address language disorders in schizophrenia: a RCT on the efficacy of a novel training targeting the pragmatics of communication (PragmaCom). J Commun Disord. 2022;97:106196. 10.1016/j.jcomdis.2022.106196. 35526293

[26] McGuiness A, Travethan L, Irvin K, Black Y, Apps J, Corinth K, et al. The potential of cognitive remediation therapy for improving the communication capabilities of adults with schizophrenia and other psychotic spectrum disorders. Int J Lang Commun Disord. 2025;60:e13141. 10.1111/1460-6984.13141. 39651878

[27] Joyal M, Bonneau A, Fecteau S. Speech and language therapies to improve pragmatics and discourse skills in patients with schizophrenia. Psychiatry Res. 2016;240:88–95. 10.1016/j.psychres.2016.04.010. 27092861

[28] Palaniyappan L, Sreeraj V, Venkatasubramanian G, Voppel A. Why is it hard to assess thought disorder? Taking stock of the third domain of psychosis. Schizophr Res. 2025;292:41–52. 10.1016/j.schres.2026.02.018 41819768

[29] Sreeraj V, Voppel A, Venkatasubramanian G, Palaniyappan L. What is being measured by formal thought disorder scales: an item-level content analysis. Psychol Med. 2025;. 10.13140/RG.2.2.24860.55682. PMC13079215 41969055

[30] Roberts LW, Warner TD, Brody JL. Perspectives of patients with schizophrenia and psychiatrists regarding ethically important aspects of research participation. AJP. 2000;157:67–74. 10.1176/ajp.157.1.67. 10618015

[31] Zullino D, Conus P, Borgeat F, Bonsack C. Readiness to participate in psychiatric research. Can J Psychiatr. 2003;48:480–4. 10.1177/070674370304800709. 12971019

[32] Humphreys K. A review of the impact of exclusion criteria on the generalizability of schizophrenia treatment research. Clin Schizophr Relat Psychoses. 2017;11:49–57. 28548580

[33] Taipale H, Schneider-Thoma J, Pinzón-Espinosa J, Radua J, Efthimiou O, Vinkers CH, et al. Representation and outcomes of individuals with schizophrenia seen in everyday practice who are ineligible for randomized clinical trials. JAMA Psychiatry. 2022;79:1–9. 10.1001/jamapsychiatry.2021.3990. PMC8792792 35080618

[34] Lawrence RE, Bernstein A, Perez Coste M, Zhao Y, Wang Y, Goldberg TE. Effectiveness trials in schizophrenia: necessary. Sufficient? Annals of Clinical Psychiatry. 2025;36:57–65. 10.1177/10401237251344105.

[35] Lakhan SE, Dorner-Ciossek C, Besedina O, Dickerson F, Hastedt C, Isla R, et al. Effectiveness, engagement, and safety of a digital therapeutic (CT-155/BI 3972080) for treating negative symptoms in people with schizophrenia: protocol for the phase 3 CONVOKE randomized controlled trial. JMIR Research Protocols. 2025;14:e81293. 10.2196/81293. 41057039 PMC12541272

[36] McCall HC, Hadjistavropoulos HD, Loutzenhiser L. Reconsidering the ethics of exclusion criteria in research on digital mental health interventions. Ethics Behav. 2021;31:171–80. 10.1080/10508422.2019.1684295.

[37] Hofer A, Hummer M, Huber R, Kurz M, Walch T, Fleischhacker WW. Selection bias in clinical trials with antipsychotics. J Clin Psychopharmacol. 2000;20:699. 11106145 10.1097/00004714-200012000-00019

[38] Patel R, Oduola S, Callard F, Wykes T, Broadbent M, Stewart R, et al. What proportion of patients with psychosis is willing to take part in research? A mental health electronic case register analysis. BMJ Open. 2017;7:e013113. 10.1136/bmjopen-2016-013113. PMC5353309 28279995

[39] Taylor JS, DeMers SM, Vig EK, Borson S. The disappearing subject: exclusion of people with cognitive impairment and dementia from geriatrics research. J Am Geriatr Soc. 2012;60:413–9. 10.1111/j.1532-5415.2011.03847.x. 22288835

[40] Lally J, Watkins R, Nash S, Shetty H, Gardner-Sood P, Smith S, et al. The representativeness of participants with severe mental illness in a psychosocial clinical trial. Front Psych. 2018;9. 10.3389/fpsyt.2018.00654. PMC6288469 30564154

[41] Kline E, Hendel V, Friedman-Yakoobian M, Mesholam-Gately RI, Findeisen A, Zimmet S, et al. A comparison of neurocognition and functioning in first episode psychosis populations: Do research samples reflect the real world? Soc Psychiatry Psychiatr Epidemiol. 2019;54:291–301. 10.1007/s00127-018-1631-x. 30488086 PMC6440832

[42] Riedel M, Strassnig M, Müller N, Zwack P, Möller H-J. How representative of everyday clinical populationsare schizophrenia patients enrolled in clinical trials? Eur Arch Psychiatry Clin Neurosci. 2005;255:143–8. 10.1007/s00406-004-0547-5. 15549345

[43] Woods SW, Ziedonis DM, Sernyak MJ, Diaz E, Rosenheck RA. Characteristics of participants and nonparticipants in medication trials for treatment of schizophrenia. PS. 2000;51:79–84. 10.1176/ps.51.1.79. 10647137

[44] Evans JD, Bond GR, Meyer PS, Kim HW, Lysaker PH, Gibson PJ, et al. Cognitive and clinical predictors of success in vocational rehabilitation in schizophrenia. Schizophr Res. 2004;70:331–42. 10.1016/j.schres.2004.01.011. 15329308

[45] Skiba RM, Chinchani AM, Menon M, Lepage M, Lavigne KM, Malla A, et al. Overlap between individual differences in cognition and symptoms of schizophrenia. Schizophr Res. 2024;270:220–8. 10.1016/j.schres.2024.06.010. 38924940

[46] Dibben CRM, Rice C, Laws K, McKenna PJ. Is executive impairment associated with schizophrenic syndromes? A meta-analysis. Psychol Med. 2009;39:381–92. 10.1017/S0033291708003887. 18588741

[47] Ventura J, Thames AD, Wood RC, Guzik LH, Hellemann GS. Disorganization and reality distortion in schizophrenia: a meta-analysis of the relationship between positive symptoms and neurocognitive deficits. Schizophr Res. 2010;121:1–14. 10.1016/j.schres.2010.05.033. 20579855 PMC3160271

[48] de Gracia Dominguez M, Viechtbauer W, Simons CJP, van Os J, Krabbendam L. Are psychotic psychopathology and neurocognition orthogonal? A systematic review of their associations. Psychol Bull. 2009;135:157–71. 10.1037/a0014415. 19210058

[49] Oeztuerk OF, Pigoni A, Antonucci LA, Koutsouleris N. Association between formal thought disorders, neurocognition and functioning in the early stages of psychosis: a systematic review of the last half-century studies. Eur Arch Psychiatry Clin Neurosci. 2022;272:381–93. 10.1007/s00406-021-01295-3. 34263359 PMC8938342

[50] McCabe R, John P, Dooley J, Healey P, Cushing A, Kingdon D, et al. Training to enhance psychiatrist communication with patients with psychosis (TEMPO): cluster randomised controlled trial. Br J Psychiatry. 2016;209:517–24. 10.1192/bjp.bp.115.179499. 27445354

[51] Sharkey RJ, Bacon C, Peterson Z, Rootes-Murdy K, Salvador R, Pomarol-Clotet E, et al. Differences in the neural correlates of schizophrenia with positive and negative formal thought disorder in patients with schizophrenia in the ENIGMA dataset. Mol Psychiatry. 2024;29:3086–96. 10.1038/s41380-024-02563-z. 38671214 PMC11449795

[52] Muroff JR, Hoerauf SL, Kim SYH. Is psychiatric research stigmatized? An experimental survey of the public. Schizophr Bull. 2006;32:129–36. 10.1093/schbul/sbj003. 16192411 PMC2632187

[53] Humphreys K, Blodgett JC, Roberts LW. The exclusion of people with psychiatric disorders from medical research. J Psychiatr Res. 2015;70:28–32. 10.1016/j.jpsychires.2015.08.005. 26424420

[54] Harris JI, Hanson D, Leskela J, Billig J, Padilla-Martinez V, Boyd J, et al. Reconsidering research exclusion for serious mental illness: ethical principles, current status, and recommendations. J Psychiatr Res. 2021;143:138–43. 10.1016/j.jpsychires.2021.09.016. 34487990

[55] Spencer BWJ, Shields G, Gergel T, Hotopf M, Owen GS. Diversity or disarray? A systematic review of decision-making capacity for treatment and research in schizophrenia and other non-affective psychoses. Psychol Med. 2017;47:1906–22. 10.1017/S0033291717000502. 28441976

[56] Spencer BWJ, Gergel T, Hotopf M, Owen GS. Unwell in hospital but not incapable: cross-sectional study on the dissociation of decision-making capacity for treatment and research in in-patients with schizophrenia and related psychoses. Br J Psychiatry. 2018;213:484–9. 10.1192/bjp.2018.85. 29909778 PMC6054873

[57] Kim SYH, Appelbaum PS, Swan J, Stroup TS, McEvoy JP, Goff DC, et al. Determining when impairment constitutes incapacity for informed consent in schizophrenia research. Br J Psychiatry. 2007;191:38–43. 10.1192/bjp.bp.106.033324. 17602123

[58] Hostiuc S, Rusu MC, Negoi I, Drima E. Testing decision-making competency of schizophrenia participants in clinical trials. A meta-analysis and meta-regression. BMC Psychiatry. 2018;18:2. 10.1186/s12888-017-1580-z. 29304845 PMC5756338

[59] Fischer BA, McMahon RP, Meyer WA, Slack DJ, Appelbaum PS, Carpenter WT. Participants with schizophrenia retain the information necessary for informed consent during clinical trials. J Clin Psychiatry. 2013;74:622–7. 10.4088/JCP.12m07997. 23842013 PMC4482016

[60] Kovnick JA, Appelbaum PS, Hoge SK, Leadbetter RA. Competence to consent to research among long-stay inpatients with chronic schizophrenia. PS. 2003;54:1247–52. 10.1176/appi.ps.54.9.1247. 12954941

[61] Howe V, Foister K, Jenkins K, Skene L, Copolov D, Keks N. Competence to give informed consent in acute psychosis is associated with symptoms rather than diagnosis. Schizophr Res. 2005;77:211–4. 10.1016/j.schres.2005.03.005. 16085206

[62] Moser DJ, Schultz SK, Arndt S, Benjamin ML, Fleming FW, Brems CS, et al. Capacity to provide informed consent for participation in schizophrenia and HIV research. Am J Psychiatry. 2002;159:1201–7. 10.1176/appi.ajp.159.7.1201. 12091200

[63] Schachter D, Kleinman I, Prendergast P, Remington G, Schertzer S. The effect of psychopathology on the ability of schizophrenic patients to give informed consent. J Nerv Ment Dis. 1994;182:360. 7911156 10.1097/00005053-199406000-00009

[64] Appelbaum PS. Decisional capacity of patients with schizophrenia to consent to research: taking stock. Schizophr Bull. 2006;32:22–5. 10.1093/schbul/sbi063. 16177275 PMC2632185

[65] Dunn LB, Candilis PJ, Roberts LW. Emerging empirical evidence on the ethics of schizophrenia research. Schizophr Bull. 2006;32:47–68. 10.1093/schbul/sbj012. 16237201 PMC2632189

[66] Palmer BW, Dunn LB, Appelbaum PS, Jeste DV. Correlates of treatment-related decision-making capacity among middle-Agedand older patients with schizophrenia. Arch Gen Psychiatry. 2004;61:230–6. 10.1001/archpsyc.61.3.230. 14993110

[67] Stroup S, Appelbaum P, Swartz M, Patel M, Davis S, Jeste D, et al. Decision-making capacity for research participation among individuals in the CATIE schizophrenia trial. Schizophr Res. 2005;80:1–8. 10.1016/j.schres.2005.08.007. 16182516

[68] Kaup AR, Dunn LB, Saks ER, Jeste DV, Palmer BW. Decisional capacity to consent to research in schizophrenia: an examination of errors. IRB. 2011;33:1–9. PMC3245677 21932481

[69] Palmer BW, Savla GN, Roesch SC, Jeste DV. Changes in capacity to consent over time in patients involved in psychiatric research. Br J Psychiatry. 2013;202:454–8. 10.1192/bjp.bp.112.121160. 23661766 PMC3669878

[70] Carpenter WT, Gold JM, Lahti AC, Queern CA, Conley RR, Bartko JJ, et al. Decisional capacity for informed consent in schizophrenia research. Arch Gen Psychiatry. 2000;57:533–8. 10839330 10.1001/archpsyc.57.6.533

[71] Silvia M-G, Georgina G, Marta F-Q, Susana O, Gemma E-R, Elena R-A, et al. RECAPACITA project: comparing neuropsychological profiles in people with severe mental disorders, with and without capacity modification. Int J Law Psychiatry. 2024;97:102035. 10.1016/j.ijlp.2024.102035. 39437608

[72] Palmer BW, Jeste DV. Relationship of individual cognitive abilities to specific components of decisional capacity among middle-aged and older patients with schizophrenia. Schizophr Bull. 2006;32:98–106. 10.1093/schbul/sbj002. 16192412 PMC2632184

[73] Stroup TS, Appelbaum PS, Gu H, Hays S, Swartz MS, Keefe RSE, et al. Longitudinal consent-related abilities among research participants with schizophrenia: results from the CATIE study. Schizophr Res. 2011;130:47–52. 10.1016/j.schres.2011.04.012. 21561740 PMC3139717

[74] Calcedo-Barba A, Fructuoso A, Martinez-Raga J, Paz S, Sánchez de Carmona M, Vicens E. A meta-review of literature reviews assessing the capacity of patients with severe mental disorders to make decisions about their healthcare. BMC Psychiatry. 2020;20:339. 10.1186/s12888-020-02756-0. 32605645 PMC7324958

[75] Georgaca E, Machaira S, Stamovlasis D, Peppou LE, Papachristou C, Arvaniti A, et al. Clinical determinants of involuntary psychiatric hospitalization: a clinical profile approach. J Clin Psychol. 2023;79:2081–100. 10.1002/jclp.23528. 37133425

[76] Monteiro LC, Silva VA, Louzã MR. Insight, cognitive dysfunction and symptomatology in schizophrenia. Eur Arch Psychiatry Clin Neurosci. 2008;258:402–5. 10.1007/s00406-008-0809-8. 18437275

[77] Zhou Y, Rosenheck R, Mohamed S, Zhang J, Chang Q, Ou Y, et al. Insight in inpatients with schizophrenia: relationship to symptoms and neuropsychological functioning. Schizophr Res. 2015;161:376–81. 10.1016/j.schres.2014.12.009. 25533592

[78] Subotnik KL, Ventura J, Hellemann GS, Zito MF, Agee ER, Nuechterlein KH. Relationship of poor insight to neurocognition, social cognition, and psychiatric symptoms in schizophrenia: a meta-analysis. Schizophr Res. 2020;220:164–71. 10.1016/j.schres.2020.03.038. 32334936

[79] Ortiz BB, Higuchi CH, Noto C, Joyce DW, Correll CU, Bressan RA, et al. A symptom combination predicting treatment-resistant schizophrenia – a strategy for real-world clinical practice. Schizophr Res. 2020;218:195–200. 10.1016/j.schres.2020.01.002. 31956005

[80] Iozzino L, Martella D, Picchioni M, Wancata J, Appelbaum PS, Mandarelli G, et al. Treatment decision making in patients with bipolar and schizophrenia spectrum disorders: a comparison. Eur Arch Psychiatry Clin Neurosci. 2025. 10.1007/s00406-025-02063-3. 40736673

[81] APPELBAUM PS. Missing the boat: competence and consent in psychiatric research. AJP. 1998;155:1486–8. 10.1176/ajp.155.11.1486. 9812106

[82] Josiassen RC, Xavier RM, Dietterich TE, Harner MK, Filmyer DM, Houpt C, et al. Obstacles, opportunities, and ethical considerations for genomic investigations of individuals continuously hospitalized with treatment-resistant schizophrenia. Schizophr Bull Open. 2025;6:sgaf019. 10.1093/schizbullopen/sgaf019. 41050266 PMC12496010

[83] Marcó-García S, Ariyo K, Owen GS, David AS. Decision making capacity for treatment in psychiatric inpatients: a systematic review and meta-analysis. Psychol Med. 2024;54:1074–83. 10.1017/S0033291724000242. 38433596

[84] Owen GS, David AS, Richardson G, Szmukler G, Hayward P, Hotopf M. Mental capacity, diagnosis and insight in psychiatric in-patients: a cross-sectional study. Psychol Med. 2008;39:1389. 10.1017/S0033291708004637. 18940026 PMC7611628

[85] Lysaker PH, Davis LW, Buck KD, Outcalt S, Ringer JM. Negative symptoms and poor insight as predictors of the similarity between client and therapist ratings of therapeutic Alliance in cognitive behavior therapy for patients with schizophrenia. J Nerv Ment Dis. 2011;199:191. 10.1097/NMD.0b013e31820c73eb. 21346490

[86] Cavelti M, Homan P, Vauth R. The impact of thought disorder on therapeutic alliance and personal recovery in schizophrenia and schizoaffective disorder: an exploratory study. Psychiatry Res. 2016;239:92–8. 10.1016/j.psychres.2016.02.070. 27137967

[87] Palmier-Claus J, Griffiths R, Murphy E, Parker S, Longden E, Bowe S, et al. Cognitive behavioural therapy for thought disorder in psychosis. Psychosis. 2017;9:347–57. 10.1080/17522439.2017.1363276.

[88] Moritz S, Woodward TS, Balzan R. Is metacognitive training for psychosis effective? Expert Rev Neurother. 2016;16(2):105–7. 26694013 10.1586/14737175.2016.1135737

[89] Fekete Z, Vass E, Balajthy R, Tana Ü, Nagy AC, Oláh B, et al. Efficacy of metacognitive training on symptom severity, neurocognition and social cognition in patients with schizophrenia: a single-blind randomized controlled trial. Scand J Psychol. 2022;63:321–33. 10.1111/sjop.12811. 35388496 PMC9544200

[90] Shryane N, Drake R, Morrison AP, Palmier-Claus J. Is cognitive behavioural therapy effective for individuals experiencing thought disorder? Psychiatry Res. 2020;285:112806. 10.1016/j.psychres.2020.112806. 32007658

[91] Fauviaux T, Parisi M, Marin L, Vattier V, Lozano-Goupil J, Mrabet D, et al. The second I laid eyes on him I knew”: first impressions predict willingness to interact with individuals with schizophrenia. Schizophr Res. 2025;283:77–84. 10.1016/j.schres.2025.06.022. 40639097

[92] Robotham D, Waterman S, Oduola S, Papoulias C, Craig T, Wykes T. Facilitating mental health research for patients, clinicians and researchers: a mixed-method study. BMJ Open. 2016;6:e011127. 10.1136/bmjopen-2016-011127. PMC4985796 27503859

[93] Jørgensen R, Munk-Jørgensen P, Lysaker PH, Buck KD, Hansson L, Zoffmann V. Overcoming recruitment barriers revealed high readiness to participate and low dropout rate among people with schizophrenia in a randomized controlled trial testing the effect of a guided self-determination intervention. BMC Psychiatry. 2014;14:28. 10.1186/1471-244X-14-28. 24490977 PMC3927263

[94] Morán-Sánchez I, Maurandi-López A, Pérez-Cárceles MD. Assessment of motivations and willingness to participate in research of outpatients with anxiety, mood, and psychotic disorders. J Empir Res Hum Res Ethics. 2018;13:546–60. 10.1177/1556264618789564. 30047823

[95] Edlinger M, Deisenhammer EA, Fiala M, Hofer A, Kemmler G, Strauss R, et al. Attitudes of patients with schizophrenia and depression towards psychiatric research. Psychiatry Res. 2010;177:172–6. 10.1016/j.psychres.2008.12.010. 20362343

[96] Kaminsky A, Roberts LW, Brody JL. Influences upon willingness to participate in schizophrenia research: an analysis of narrative data from 63 people with schizophrenia. Ethics Behav. 2003;13:279–302. 10.1207/S15327019EB1303_06. 14680009

[97] Roberts LW, Warner TD, Hammond KG, Hoop JG. Views of people with schizophrenia regarding aspects of research: study size and funding sources. Schizophr Bull. 2006;32:107–15. 10.1093/schbul/sbj022. 16254065 PMC2632193

[98] Dunn LB, Palmer BW, Keehan M, Jeste DV, Appelbaum PS. Assessment of therapeutic misconception in older schizophrenia patients with a brief instrument. AJP. 2006;163:500–6. 10.1176/appi.ajp.163.3.500. 16513873

[99] Candilis PJ, Geppert CMA, Fletcher KE, Lidz CW, Appelbaum PS. Willingness of subjects with thought disorder to participate in research. Schizophr Bull. 2006;32:159–65. 10.1093/schbul/sbj016. 16254062 PMC2632177

[100] Wu B-J, Liao H-Y, Chen H-K, Lan T-H. Psychopathology, psychopharmacological properties, decision-making capacity to consent to clinical research and the willingness to participate among long-term hospitalized patients with schizophrenia. Psychiatry Res. 2016;237:323–30. 10.1016/j.psychres.2016.01.020. 26847945

[101] Dunn LB, Kim DS, Fellows IE, Palmer BW. Worth the risk? Relationship of incentives to risk and benefit perceptions and willingness to participate in schizophrenia research. Schizophr Bull. 2009;35:730–7. 10.1093/schbul/sbn003. 18281293 PMC2696364

[102] Roberts LW, Dunn LB, Green Hammond KA, Warner TD. Do research procedures pose relatively greater risk for healthy persons than for persons with schizophrenia? Schizophr Bull. 2006;32:153–8. 10.1093/schbul/sbi055. 16166609 PMC2632188

[103] Roberts LW, Warner TD, Brody JL, Roberts B, Lauriello J, Lyketsos C. Patient and psychiatrist ratings of hypothetical schizophrenia research protocols: assessment of harm potential and factors influencing participation decisions. AJP. 2002;159:573–84. 10.1176/appi.ajp.159.4.573. 11925295

[104] Kim JP, Ryan K, Tsungmey T, Kasun M, Roberts WA, Dunn LB, et al. Perceived protectiveness of research safeguards and influences on willingness to participate in research: a novel MTurk pilot study. J Psychiatr Res. 2021;138:200–6. 10.1016/j.jpsychires.2021.04.005. 33865169 PMC8513533

[105] Roberts LW, Kim JP, Tsungmey T, Dunn LB. Do human subject safeguards matter to potential participants in psychiatric genetic research? J Psychiatr Res. 2019;116:95–103. 10.1016/j.jpsychires.2019.06.004. 31226581 PMC6703554

[106] Roberts LW, Warner TD, Anderson CT, Smithpeter MV, Rogers MK. Schizophrenia research participants’ responses to protocol safeguards: recruitment, consent, and debriefing. Schizophr Res. 2004;67:283–91. 10.1016/S0920-9964(03)00101-4. 14984889

[107] Eadie TL, Yorkston KM, Klasner ER, Dudgeon BJ, Deitz JC, Baylor CR, et al. Measuring communicative participation: a review of self-report instruments in speech-language pathology. Am J Speech Lang Pathol. 2006;15:307–20. 10.1044/1058-0360(2006/030. 17102143 PMC2649949

[108] Langdon R, Coltheart M, Ward PB, Catts SV. Disturbed communication in schizophrenia: the role of poor pragmatics and poor mind-reading. Psychol Med. 2002;32:1273–84. 10.1017/S0033291702006396. 12420896

[109] Delgaram-Nejad O, Archer D, Chatzidamianos G, Robinson L, Bartha A. The DAIS-C: a small, specialised, spoken, schizophrenia corpus. Applied Corpus Linguistics. 2023;3:100069. 10.1016/j.acorp.2023.100069.

[110] Çokal D, Zimmerer V, Varley R, Watson S, Hinzen W. Comprehension of embedded clauses in schizophrenia with and without formal thought disorder. J Nerv Ment Dis. 2019;207:384. 10.1097/NMD.0000000000000981. 30958421

[111] Tan EJ, Yelland GW, Rossell SL. Characterising receptive language processing in schizophrenia using word and sentence tasks. Cogn Neuropsychiatry. 2016;21:14–31. 10.1080/13546805.2015.1121866. 27031118

[112] Tan EJ, Rossell SL. Language comprehension and neurocognition independently and concurrently contribute to formal thought disorder severity in schizophrenia. Schizophr Res. 2019;204:133–7. 10.1016/j.schres.2018.08.019. 30126817

[113] Rossetti I, Brambilla P, Papagno C. Metaphor comprehension in schizophrenic patients. Front Psychol. 2018;9. 10.3389/fpsyg.2018.00670. 29867648 PMC5954116

[114] Sharpe V, Schoot L, Lewandowski KE, Öngür D, Türközer HB, Hasoğlu T, et al. We both say tomato: intact lexical alignment in schizophrenia and bipolar disorder. Schizophr Res. 2022;243:138–46. 10.1016/j.schres.2022.02.032. 35290874 PMC9188992

[115] Bambini V, Arcara G, Bosinelli F, Buonocore M, Bechi M, Cavallaro R, et al. A leopard cannot change its spots: a novel pragmatic account of concretism in schizophrenia. Neuropsychologia. 2020;139:107332. 10.1016/j.neuropsychologia.2020.107332. 31923528

[116] Elleuch D, Chen Y, Luo Q, Palaniyappan L. Relationship between grammar and schizophrenia: a systematic review and meta-analysis. Commun Med (Lond). 2025;5:235. 10.1038/s43856-025-00944-1. 40523895 PMC12170843

[117] Docherty NM, Evans IM, Sledge WH, Seibyl JP, Krystal JH. Affective reactivity of language in schizophrenia. J Nerv Ment Dis. 1994;182:98–103. 8308539 10.1097/00005053-199402000-00006

[118] Zaher F, Diallo M, Achim AM, Joober R, Roy M-A, Demers M-F, et al. Speech markers to predict and prevent recurrent episodes of psychosis: a narrative overview and emerging opportunities. Schizophr Res. 2024;266:205–15. 10.1016/j.schres.2024.02.036. 38428118

[119] de Winter L, Vermeulen JM, Couwenbergh C, van Weeghel J, Hasson-Ohayon I, Mulder CL, et al. Short- and long-term changes in symptom dimensions among patients with schizophrenia spectrum disorders and different durations of illness: a meta-analysis. J Psychiatr Res. 2023;164:416–39. 10.1016/j.jpsychires.2023.06.031. 37429186

[120] Rønne ST, Arnfred SM, Gæde PH, Cleal B, Jørgensen R. Recruiting underrepresented populations for surveys: the case of people with schizophrenia and coexisting diabetes. Nord J Psychiatry. 2025;79:333–8. 10.1080/08039488.2025.2502932. 40390336

[121] Jones H, Cipriani A. Barriers and incentives to recruitment in mental health clinical trials. Evid Based Mental Health. 2019;22. 10.1136/ebmental-2019-300090. PMC10270385 31023822

[122] Lipsky M. Street level bureaucracy: dilemmas of the individual in public services. Russell Sage Foundation; 1980.

[123] Gillies K, Duthie A, Cotton S, Campbell MK. Patient reported measures of informed consent for clinical trials: a systematic review. PLoS One. 2018;13:e0199775. 10.1371/journal.pone.0199775. 29949627 PMC6021104

[124] Dunn LB, Nowrangi MA, Palmer BW, Jeste DV, Saks ER. Assessing decisional capacity for clinical research or treatment: a review of instruments. AJP. 2006;163:1323–34. 10.1176/ajp.2006.163.8.1323. 16877642

[125] Flory J, Emanuel E. Interventions to improve research participants’ understanding in informed consent for research: a systematic review. JAMA. 2004;292:1593–601. 10.1001/jama.292.13.1593. 15467062

[126] Harmell AL, Palmer BW, Jeste DV. Preliminary study of a web-based tool for enhancing the informed consent process in schizophrenia research. Schizophr Res. 2012;141:247–50. 10.1016/j.schres.2012.08.001. 22939457 PMC3471544

[127] Kim SYH, Caine ED, Swan JG, Appelbaum PS. Do clinicians follow a risk-sensitive model of capacity-determination? An experimental video survey. Psychosomatics. 2006;47:325–9. 10.1176/appi.psy.47.4.325. 16844891

[128] Kim SYH, Appelbaum PS, Kim HM, Wall IF, Bourgeois JA, Frankel B, et al. Variability of judgments of capacity: experience of capacity evaluators in a study of research consent capacity. Psychosomatics. 2011;52:346–53. 10.1016/j.psym.2011.01.012. 21777717 PMC3142349

[129] Di Fazio N, Morena D, Piras F, Piras F, Banaj N, Delogu G, et al. Reliability of clinical judgment for evaluation of informed consent in mental health settings and the validation of the evaluation of informed consent to treatment (EICT) scale. Front Psychol. 2024;15. 10.3389/fpsyg.2024.1309909. PMC10986368 38566948

[130] Weissinger GM, Ulrich CM. Informed consent and ethical reporting of research in clinical trials involving participants with psychotic disorders. Contemp Clin Trials. 2019;84:105795. 10.1016/j.cct.2019.06.009. 31247285 PMC11520818

[131] Jeste DV, Palmer BW, Appelbaum PS, Golshan S, Glorioso D, Dunn LB, et al. A new brief instrument for assessing decisional capacity for clinical research. Arch Gen Psychiatry. 2007;64:966–74. 10.1001/archpsyc.64.8.966. 17679641

[132] Hamilton RKB, Phelan CH, Chin NA, Wyman MF, Lambrou N, Cobb N, et al. The U-ARE protocol: a pragmatic approach to decisional capacity assessment for clinical research. J Alzheimer’s Dis. 2020;73:431–42. 10.3233/JAD-190457. 31868663 PMC7388558

[133] Dunn LB. Capacity to consent to research in schizophrenia: the expanding evidence base. Behav Sci Law. 2006;24:431–45. 10.1002/bsl.698. 16883608

[134] Appelbaum PS, Grisso T. Assessing patients’ capacities to consent to treatment. N Engl J Med. 1988;319:1635–8. 10.1056/NEJM198812223192504. 3200278

[135] Jeste DV, Palmer BW, Golshan S, Eyler LT, Dunn LB, Meeks T, et al. Multimedia consent for research in people with schizophrenia and Normal subjects: a randomized controlled trial. Schizophr Bull. 2009;35:719–29. 10.1093/schbul/sbm148. 18245061 PMC2696362

[136] Dunn LB, Lindamer LA, Palmer BW, Schneiderman LJ, Jeste DV. Enhancing comprehension of consent for research in older patients with psychosis: a randomized study of a novel consent procedure. Am J Psychiatry. 2001;158:1911–3. 10.1176/appi.ajp.158.11.1911. 11691700

[137] Palmer BW, Cassidy EL, Dunn LB, Spira AP, Sheikh JI. Effective use of consent forms and interactive questions in the consent process. IRB: Ethics & Human Research. 2008;30:8–12. 18512654

[138] Stiles PG, Poythress NG, Hall A, Falkenbach D, Williams R. Improving understanding of research consent disclosures among persons with mental illness. PS. 2001;52:780–5. 10.1176/appi.ps.52.6.780. 11376225

[139] DuBois JM, Bante H, Hadley WB. Ethics in psychiatric research: a review of 25 years of NIH-funded empirical research projects. AJOB Prim Res. 2011;2:5–17. 10.1080/21507716.2011.631514. 23259152 PMC3524581

[140] Dunn LB, Jeste DV. Enhancing informed consent for research and treatment. Neuropsychopharmacol. 2001;24:595–607. 10.1016/S0893-133X(00)00218-9. 11331139

[141] Hodgins V, Mouslih CE, Rukh-E-Qamar H, Titone D. Multilingualism and psychosis: a pre-registered scoping review. Biling Lang Congn. 2025;28:846–56. 10.1017/S1366728924000890.

[142] Mathew J. Readability of consent forms in schizophrenia research. Aust N Z J Psychiatry. 2002;36:564–5. 10.1046/j.1440-1614.2002.t01-8-01066.x. 12169166

[143] Schizophrenia International Research Society. Enhancing informed consent in schizophrenia research by harnessing the power of lived experience perspectives. Available at: https://schizophreniaresearchsociety.org/enhancing-informed-consent-in-schizophrenia-research-by-harnessing-the-power-of-lived-experience-perspectives/ (accessed 26 October 2025).

[144] Flesch R. A new readability yardstick. J Appl Psychol. 1948;32:221–33. 10.1037/h0057532. 18867058

[145] Kim SYH, Karlawish JH, Kim HM, Wall IF, Bozoki AC, Appelbaum PS. Preservation of the capacity to appoint a proxy decision maker: implications for dementia research. Arch Gen Psychiatry. 2011;68:214–20. 10.1001/archgenpsychiatry.2010.191. 21300949 PMC3349937

[146] Stroup S, Appelbaum P. The subject advocate: protecting the interests of participants with fluctuating Decisionmaking capacity. IRB: Ethics & Human Research. 2003;25:9–11. 10.2307/3564299. 14569988

[147] Hodgins V, O’Driscoll G, Titone D. The impact of neurotypical cognition on communication deficits attributed to pathologized people: schizophrenia as a case study. Appl Psycholinguist. 2023;44:330–42. 10.1017/S0142716422000340.

[148] Kopelowicz A, Liberman RP, Zarate R. Recent advances in social skills training for schizophrenia. Schizophr Bull. 2006;32:S12–23. 10.1093/schbul/sbl023. 16885207 PMC2632540

[149] Keith Massel H, Corrigan PW, Liberman RP, Milan MA. Conversation skills training of thought-disordered schizophrenic patients through attention focusing. Psychiatry Res. 1991;38:51–61. 10.1016/0165-1781(91)90052-Q. 1946834

[150] Wong SE, Martinez-Diaz JA, Massel HK, Edelstein BA, Wiegand W, Bowen L, et al. Conversational skills training with schizophrenic inpatients: a study of generalization across settings and conversants. Behav Ther. 1993;24:285–304. 10.1016/S0005-7894(05)80270-9.

[151] Wong SE, Woolsey JE. Re-establishing conversational skills in overtly psychotic, chronic schizophrenic patients: discrete trials training on the psychiatric Ward. Behav Modif. 1989;13:415–30. 10.1177/01454455890134002. 2818460

[152] Penn C, Frankel T, Watermeyer J, Müller M. Informed consent and aphasia: evidence of pitfalls in the process. Aphasiology. 2009;23:3–32. 10.1080/02687030701521786.

[153] Stein J, Brady Wagner LC. Is informed consent a “yes or no” response? Enhancing the shared decision-making process for persons with aphasia. Top Stroke Rehabil. 2006;13:42–6. 10.1310/tsr1304-42. 17082168

[154] Beck KB, MacKenziem KT, Kirby AV, McDonald K, Moura I, Breitenfeldt K, et al. Guidelines for the creation of accessible consent materials and procedures: lessons from research with autistic people and people with intellectual disability. Autism Adulthood. 2025;. 10.1089/aut.2024.0263. PMC12448064 40979614

[155] Pilnick A, Trusson D, Beeke S, O’Brien R, Goldberg S, Harwood RH. Using conversation analysis to inform role play and simulated interaction in communications skills training for healthcare professionals: identifying avenues for further development through a scoping review. BMC Med Educ. 2018;18:267. 10.1186/s12909-018-1381-1. 30453956 PMC6245918

[156] Golay P, Baumann PS, Jenni R, Do KQ, Conus P. Patients participating to neurobiological research in early psychosis: a selected subgroup? Schizophr Res. 2018;201:249–53. 10.1016/j.schres.2018.04.033. 29705004

[157] Howard L, de Salis I, Tomlin Z, Thornicroft G, Donovan J. Why is recruitment to trials difficult? An investigation into recruitment difficulties in an RCT of supported employment in patients with severe mental illness. Contemp Clin Trials. 2009;30:40–6. 10.1016/j.cct.2008.07.007. 18718555 PMC2626649

[158] Yoon J, Mayer MR, Berro T, Brazis S, Kantrowitz JT. Knowing is half the Battle: the factors leading to efficient recruitment of representative samples in schizophrenia research. Pharm Med. 2025;. 10.1007/s40290-024-00545-8. 39794624

[159] Deckler E, Ferland M, Brazis S, Mayer MR, Carlson M, Kantrowitz JT. Challenges and strategies for the recruitment of patients with schizophrenia in a research setting. Int J Neuropsychopharmacol. 2022;25:924–32. 10.1093/ijnp/pyac058. 36037521 PMC9452184

[160] Tranberg K, Due TD, Rozing M, Jønsson ABR, Kousgaard MB, Møller A. Challenges in reaching patients with severe mental illness for trials in general practice—a convergent mixed methods study based on the SOFIA pilot trial. Pilot and Feasibility Studies. 2023;9:182. 10.1186/s40814-023-01395-y. 37908003 PMC10617218

[161] Callard F, Broadbent M, Denis M, Hotopf M, Soncul M, Wykes T, et al. Developing a new model for patient recruitment in mental health services: a cohort study using electronic health records. BMJ Open. 2014;4:e005654. 10.1136/bmjopen-2014-005654. PMC4256538 25468503

[162] Bosco FM, Gabbatore I, Gastaldo L, Sacco K. Communicative-pragmatic treatment in schizophrenia: a pilot study. Front Psychol. 2016;7. 10.3389/fpsyg.2016.00166. 26941667 PMC4762993

[163] Jones N, Atterbury K, Byrne L, Carras M, Brown M, Phalen P. Lived experience, research leadership, and the transformation of mental health services: building a researcher pipeline. PS. 2021;72:591–3. 10.1176/appi.ps.202000468. 33691492

[164] Molloy N, Kilcoyne I, Belcher H, Wykes T. Exploring the involvement of people with lived experience of mental disorders in co-developing outcome measures: a systematic review. Lancet Psychiatry. 2025;12:140–52. 10.1016/S2215-0366(24)00376-6. 39848731

[165] Bora E, Yalincetin B, Akdede BB, Alptekin K. Neurocognitive and linguistic correlates of positive and negative formal thought disorder: a meta-analysis. Schizophr Res. 2019;209:2–11. 10.1016/j.schres.2019.05.025. 31153670

[166] Elleuch D, Chen Y, Luo Q, Palaniyappan L Syntax and Schizophrenia: A meta-analysis of comprehension and production 2024. 10.1101/2024.10.26.24316171.

[167] Cavelti M, Kircher T, Nagels A, Strik W, Homan P. Is formal thought disorder in schizophrenia related to structural and functional aberrations in the language network? A systematic review of neuroimaging findings. Schizophr Res. 2018;199:2–16. 10.1016/j.schres.2018.02.051. 29510928

[168] Sumner PJ, Bell IH, Rossell SL. A systematic review of task-based functional neuroimaging studies investigating language, semantic and executive processes in thought disorder. Neurosci Biobehav Rev. 2018;94:59–75. 10.1016/j.neubiorev.2018.08.005. 30142368

[169] Goldsmith LP, Morshead R, McWilliam C, Forbes G, Ussher M, Simpson A, et al. Co-producing randomized controlled trials: how Do we work together? Front Sociol. 2019;4. 10.3389/fsoc.2019.00021. PMC8022576 33869347

[170] Charlton JI. Nothing about us without us: disability oppression and empowerment. 1st ed. University of California Press; 1998.

[171] Hoekstra RA, Girma F, Tekola B, Yenus Z. Nothing about us without us: the importance of local collaboration and engagement in the global study of autism. BJPsych Int. 2018;15:40–3. 10.1192/bji.2017.26. 29953134 PMC6020913

[172] Jones N, Callejas L, Brown M, Carras MC, Croft B, Pagdon S, et al. Barriers to meaningful participatory mental health services research and priority next steps: findings from a National Survey. Psychiatr Serv. 2023;74:902–10. 10.1176/appi.ps.20220514. 36935620 PMC11022526

