# Supplementary material for: Increasing participation of people with thought disorder in clinical research
Source: Eur Psychiatry. 2026 Apr 27;69(1):e56. doi: 10.1192/j.eurpsy.2026.12211 (PMC13227140; doi:10.1192/j.eurpsy.2026.12211)
Supplement: Palaniyappan et al. supplementary material [file S0924933826122111sup001.docx]

## Supplement

**Supplement 1**

An example describing how research data will be handled in a research study focused on thought disorder. This was developed in partnership with lived experience experts, with reading ease improved using Microsoft’s Copilot.

| **Questions on data handling** | **A sample of answers with Flesch-Kincaid Grade level 7.3** |
| --- | --- |
| What kind of information is collected? | A recording of what you say in a 20- minute conversation, pictures of your brain and a count of your symptoms. |
| Who will get to see this information? | The people in charge listed in the consent form and the research staff approved by them. |
| How will the information be kept safe? | Your data will be stored securely at the Douglas Research Centre at McGill University. First, we will remove your name and other details. Then, we will transfer the data using safe methods and store it with a password. |
| Will my personal details be shared? | Your personal details (name, address, birthdate) will not be shared with researchers outside Douglas Research Centre. |
| Can I decide to leave the study in the future? | Yes, you can leave the study anytime. You can let us use the data we collected before you leave, but you don't have to. |
| Will the information be used to make money? | We will use your data to teach and learn more about this research. We will not give it to companies or individuals who will make money from it. |
| What are the health benefits for me? | You may not get any personal health benefits from being in this study. |

**Supplement 2**

An Example of Readability: Comparing Easy vs. Fairly Difficult Texts Using Flesch-Kincaid Scores

| Flesch-Kincaid Grade Level: 11.4  Flesch Reading Ease Score: 55.9  <https://www.mcgill.ca/bic/files/bic/icf_megexample_eng.docx> | **Magnetoencephalography (MEG):** MEG measures the magnetic fields generated by the tiny electrical currents that flow in your brain. The measures are performed without injecting any compound in your body, and without impressing external energy or radiation. The MEG instrument is just sensitive to very small magnetic fields and is able to pick up activity from your brain. Because it is so sensitive, you and the instrument must be in a quiet environment, where there are few electromagnetic waves generated by computers, cell phones, traffic, etc. You will therefore be required to stay in a special room during the test. This room is made of several layers of metal that are very good at weakening the electromagnetic perturbations from outside that could affect the quality of the data. You will also be asked to change into scrubs or a hospital gown to make sure your clothes or particles on their fabric do not alter data quality: you will be able to change in a private changing room or bathroom near the MEG suite.  The MEG operator will tape a few electrodes on your torso (for monitoring heartbeats) and on your forehead and cheeks (for monitoring eye movements): this is to control for normal magnetic fluctuations generated by eye movements and the heart. The operator will also tape about 4 small coils on your head to monitor your head position inside the MEG instrument. Finally, the operator will use a pencil-like device to model the shape of your head in 3 dimensions. This allows to align MEG results with MRI images.  During the test, you will be asked to either sit on a chair or lie on a bed. In either case, only the top, back and sides of your head will be covered by the MEG helmet. At all times, you will be able to communicate by talking with the MEG operator who will be sitting immediately outside the MEG room. The operator will also be able to see you, through a video system. If you do not feel comfortable at any moment during the test, please speak up, wave your hands or simply stand-up and walk out of the room: you will be able to open the MEG room door from the inside. If you think you would feel more comfortable in the MEG room with somebody else sitting next to you, it is entirely possible, and you should simply inform the investigator. |
| --- | --- |
| Flesch-Kincaid Grade Level: 4.5  Flesch Reading Ease Score: 84.5 | **Getting an MEG Brain Scan**  An MEG scan is a way for researchers to record your brain's magnetic signals. It is safe because nothing is injected into your body, you do not need to ingest any chemicals to prepare for the scan, and it does not use any radiation.  Because the machine is super sensitive, the test is done in a special quiet room. This room is built like a fortress to block out magnetic waves from things like computers, phones, and cars outside.  **Before the test:**  You will change into hospital clothes (called scrubs) in a private room. This is to make sure no metal bits on your regular clothes mess up the scan.  The technician will place a few stickers on your body:  A few on your chest to track your heartbeat.  A few on your face to track your eye blinks and movements.  A few small coils on your head so the machine knows where your head is.  **During the test:**  You will either sit in a chair or lie on a bed.  The machine looks like a large helmet or hair dryer. It will go over the top and sides of your head, but your face is never covered.  You can talk to the technician at any time. They will be right outside the room watching you on a video screen.  **Important**: If you feel uncomfortable or need to stop, just say so, wave your hands, or stand up. You can always open the door and walk out.  If you want a friend or family member to sit in the room with you, just ask! The technicians are there to make sure you are comfortable. |

A video guide to Magnetoencephalography (MEG) can be found here: <https://www.youtube.com/watch?v=sE3m25LzztY>.
This video explains what a MEG scan is and what happens during one. It covers the basics of a MEG scan and details of where you will get scanned and how you will be prepared for the scan.

**Supplement 3**Strategies to enhance inclusion of participants with thought disorder across recruitment, consent, and participation phases
 **
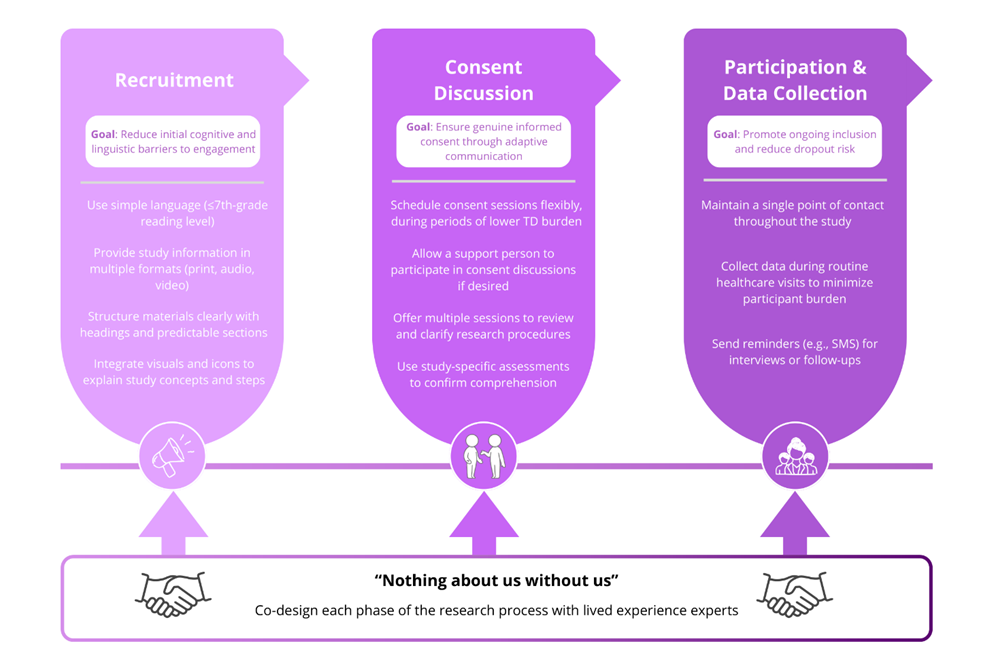
**
